# Web-Based Cognitive Bias Intervention for Psychiatric Disorders: Protocol for a Systematic Review

**DOI:** 10.2196/10427

**Published:** 2018-08-07

**Authors:** Melvyn Zhang, Jiangbo Ying, Guo Song, Daniel SS Fung, Helen Smith

**Affiliations:** ^1^ National Addictions Management Service Institute of Mental Health Singapore Singapore; ^2^ Department of Developmental Psychiatry Institute of Mental Health Singapore Singapore; ^3^ Family Medicine and Primary Care Lee Kong Chian School of Medicine Nanyang Technological University Singapore Singapore Singapore

**Keywords:** cognitive bias modification, attention bias modification, psychology, psychiatry, internet, eHealth

## Abstract

**Background:**

Traditional psychological therapies focus mainly on modification of individuals’ conscious decision-making process. Unconscious processes, such as cognitive biases, have been found accountable for various psychiatric psychopathologies, and advances in technologies have transformed how bias modification programs are being delivered.

**Objective:**

The primary aim of this review is to synthesize evidence of Web-based cognitive bias modification intervention for bias reduction. The secondary aim is to determine the change in symptoms for individual psychiatric disorders following bias modification.

**Methods:**

A systematic review will be conducted including only randomized trials. There will be no restrictions on participants included in the study. A search will be conducted on the respective databases until 2017. Selection of studies will be by the Preferred Reporting Items for Systematic Review and Meta-Analyses (PRISMA-P) guidelines. Quality assessment of included studies will be conducted using the Cochrane Risk of Bias tool. A narrative synthesis of identified articles will then be conducted. A meta-analysis will be considered only if there are sufficient articles in a domain for statistical analysis. Ethical approval for this protocol and the planned systematic review was not required.

**Results:**

We expect that the review will be completed 12 months from publication of this protocol.

**Conclusions:**

This review is of importance given how technology has transformed delivery of conventional therapies. Findings from this review will guide future research involving technology and cognitive bias modification interventions.

**Trial Registration:**

International Prospective Register for Systematic Reviews (PROSPERO): 2017 CRD42017074754; https://www.crd.york.ac.uk/prospero/display_record.php?RecordID=74754 (Archived by WebCite at http://www.webcitation.org/ 71AvSgZGn)

**Registered Report Identifier:**

RR1-10.2196/10427

## Introduction

Advances in experimental psychology over the last decade have led to growing interest and further research in cognitive bias and cognitive bias modification. Cognitive bias includes attentional biases, approach bias, and interpretative biases [1], and the presence of these automatic biases results in individuals attending to or approaching certain stimuli in their environment or making negative interpretations when faced with ambiguity [2]. For instance, individuals with substance use disorders would attend to and approach substance-related cues and items in their natural environment more readily because of the presence of these automatic processes, and this might be responsible for them relapsing. Cognitive bias is involved in psychopathologies of several psychiatric disorders, ranging from social anxiety disorder [3] to alcohol [4] and tobacco use disorders [5]. Findings that these biases are present led to bias modification intervention to retrain these automatic attentional approach tendencies or interpretational tendencies toward certain stimuli [2]. These interventions are commonly referred to generally as cognitive bias modification. Attentional bias retraining involves use of either the dot-probe or visual probe task, and this involves pairing the probe with the neutral word or image all the time [6]. Approach biases retraining involves the use of the approach or avoidance task, which includes having the individual push away substance-related cues [7]. Interpretative bias retraining involves training participants to make positive interpretations using ambiguous scenarios or the word sentence association task [8].

To date, apart from increased recognition of cognitive biases and understanding of underlying theoretical approaches, several studies have examined the effectiveness of cognitive bias modification for various psychiatric disorders. Manning et al (2016) [9] reported the feasibility of delivering cognitive bias modification, targeting approach biases for inpatient individuals attending an alcohol detoxification program. They [9] also reported that cognitive bias modification aided individuals in maintenance of abstinence. They [9] reported no differences between experimental and control groups regarding secondary outcomes, such as the craving score, time to relapse, mean drinking days, and mean drinks per drinking day. Regarding performance on the bias modification task, participants’ response to the task became faster with more sessions completed. Cognitive bias modification therapy has been investigated in other addictive disorders, such as that of cocaine use disorder [10]. Cognitive bias modification has also been evaluated among individuals with other psychiatric disorders, depressive disorders [11], and anxiety disorders [12]. Recent studies have attempted to synthesize the evidence base for cognitive bias modification for various disorders. Cristea et al’s (2016) [13] prior meta-analytic review reported that cognitive bias modification was effective in reducing cognitive biases for participants with alcohol or tobacco use disorders. They reported that bias modification for both attentional and approach biases was moderately effective with an effect size of 0.60 (Hedge G) [13]. However, they also reported that changes in attentional biases did not lead to changes in symptomatology, such as cravings and other addiction outcomes. Jones et al’s (2017) [2] review of meta-analysis reported that cognitive bias modification was most effective for anxiety disorders. Jones et al (2017) reported that among 10 studies they identified for anxiety disorder, 8 reported significant results. The effect size for anxiety disorders ranged from 0.13 to 0.74 [2]. They also reported that only 3 of 7 studies provided evidence for cognitive bias modification of depressive symptoms with effect sizes ranging from 0.35 to 0.85 [2]. They also reported that cognitive bias modification was effective (based on results from 2 meta-analyses) for eating disorders, smoking, and alcohol use with effect sizes ranging from 0.003 to 0.36 [2]. Thus, published studies to date have demonstrated the potential of cognitive bias modification in targeting cognitive biases intrinsic to various psychiatric disorders.

One of the key findings arising from Jones et al’s (2017) [2] review was that contextual factors do influence the overall effectiveness of interventions. Although Jones et al’s (2017) [2] prior review reported that cognitive bias paradigms are most efficacious when administered in a laboratory environment, there remains to date quite a number of cognitive bias modification interventions administered remotely. The increasing number of remote Web-based therapies has largely been attributed to major advances in eHealth technologies; eHealth is defined as the process in which health resources and health care are communicated and transferred by an electronic medium, and it includes Web-based interventions as well as telephone-delivered therapy and texting [14]. Since the advent of eHealth, various medical disciplines have evaluated these technologies for self-management of chronic diseases, thus facilitating individuals’ participation in rehabilitation programs and supporting outreach efforts in rural areas [15]. Web-based technologies are widely utilized in mental health for delivery of various forms of psychological therapy, and they have been proven efficacious for disorders such as depression and anxiety. However, the growth of Web-based interventions is not limited to existing therapies. An increasing number of studies have examined the efficacy of Web-based cognitive bias interventions. Attentional control training and approach bias retraining were administered using a Web-based program to 136 problem drinkers in Wiers et al’s (2015) [16] previous study. Similarly, cognitive bias modification targeting imagery and interpretation bias were administered to participants with depressive disorder using a Web-based program in Blackwell et al’s (2015) [17] previous study. There are several distinct advantages of delivering cognitive bias modification via the Web. Use of web technologies removes geographical barriers that would traditionally hinder participants from or limit them to receiving such interventions [18]. Participants are also no longer confined in a laboratory environment and can undertake the same intervention in the comfort of their home environment [17]. Web-based interventions also remove the need for a therapist, and this will facilitate dissemination of the intervention to multiple individuals [19].

In their meta-meta-analyses, Jones et al (2017) [2] have included 2 meta-analytical articles [20,21] that have synthesized evidence for Web-based cognitive bias modification interventions. Both meta-analyses focused on the technology applied to cognitive bias modification for anxiety disorders. Also, only 4 of the studies included in the Price et al (2016) meta-analysis were delivered in the home environment with the latest study published in 2014 [21]. For Kampmann et al’s (2016) meta-analysis [20], only 7 studies delivered bias interventions outside a laboratory environment with the latest study published in 2015. For social anxiety disorder, there have been further evaluations, such as that by Hullu et al (2017) [22]. There has also been further evaluation of Web-based interventions for other disorders, such as for anxiety and depressive symptoms in adolescents [23,24,25] and for addictive disorders [19,26,27]. Given further studies for anxiety disorders and potentially more studies evaluating Web-based cognitive bias modification in other disorders, this review is crucial and pertinent. Understanding the efficacy of Web-based cognitive bias interventions would guide further research that seeks to harness technology in the delivery of cognitive bias modification interventions.

The primary aim of this review is to synthesize the evidence of Web-based cognitive bias modification intervention for bias reduction. The secondary aim is to determine the change in symptoms for individual psychiatric disorders following bias modification.

## Methods

### Search Strategy

To identify relevant articles, the following search terminologies will be used:

(“Cognitive bias” OR “attention bias” OR “approach bias” or “interpretative bias”) AND (“Internet” OR “Web” OR “Online”). Search terms within each concept will be combined using the Boolean operator “OR” and search terms between 2 disparate concepts will be combined using the Boolean operator “AND.”

A comprehensive search will be conducted on PubMed, MEDLINE, EMBASE, PsycINFO, Science Direct, Cochrane CENTRAL, and Scopus databases. If full-text access is not available, original authors will be contacted. Proceedings from scientific meetings and conference abstracts will also be included.

### Inclusion and Exclusion Criteria

Only articles in the English language will be included. Inclusion criteria are the condition examined must be a psychiatric disorder, the diagnosis must be made either by a structured clinical interview or by means of a questionnaire, cognitive bias modification must have been delivered using a Web-based modality, the study design is that of a randomized trial, and Web-based cognitive bias modification intervention must be delivered as a standalone (not in conjunction with other modalities of therapy).

Exclusion criteria are the intervention did not include a validated measure for attention bias or cognitive bias assessment, the intervention was delivered by a mobile phone device, the intervention was delivered by a mobile game, attention bias modification is part of a pharmacological trial, and Web-based cognitive bias modification is delivered in conjunction with other therapies

### Condition or Domains Studied

This review seeks to determine the effectiveness of Web-based cognitive bias modification in psychiatric disorders.

### Participants

Adult, child, and adolescent populations will be considered for inclusion in this systematic review. There are no restrictions on participants included in these studies. Participants could be either individuals from the general population or a treatment-seeking cohort.

### Intervention and Exposure

The intervention is that of a Web-based cognitive bias modification task. This might include attention bias modification, cognitive bias modification for interpretation, and a visual search or spatial cueing modification task.

### Comparator

For randomized trials, we will determine whether participants are compared against individuals who have received placebo training or a sham training intervention.

### Outcome

For the primary outcome, we will report whether cognitive bias modification has been effective, which is defined as reduction in biases following intervention. For secondary outcomes, we will report whether there are reductions in overall anxiety and depression symptoms, as measured by validated questionnaires, or for addictive disorders, whether there are reductions in overall craving, reductions in total amount of substance consumed, and increases in total duration of abstinence. For secondary outcomes, scores on questionnaires administered pre- and postintervention will be reported.

### Data Extraction, Sorting, and Selection

**Figure 1:**
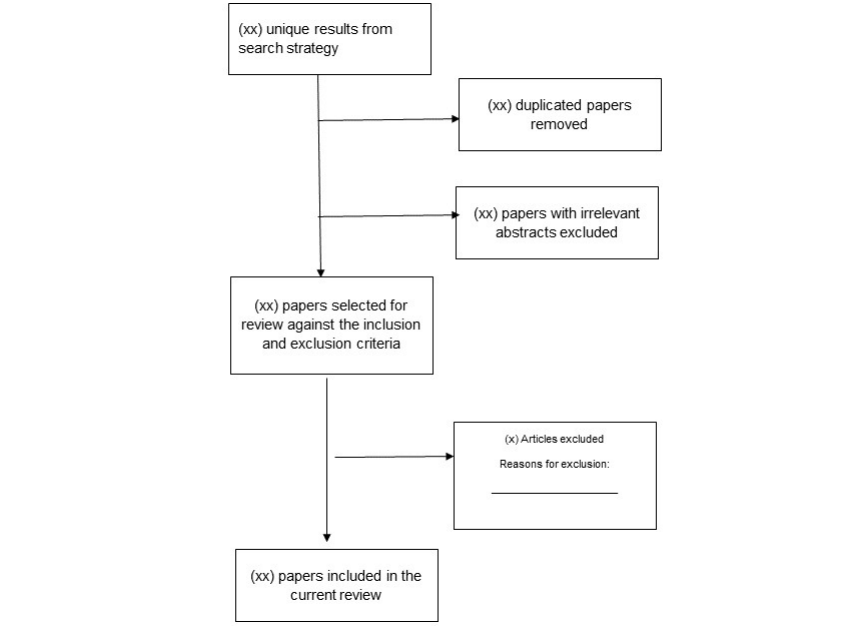
Sample PRISMA flowchart.

Database searches will be conducted, and abstracts will be obtained and imported in the reference manager Endnote X8. The first author (MWBZ) will review the compiled database and will remove all duplicated articles. Articles will be further independently screened by authors (MWBZ and JY) based on their titles and abstracts. The full text of shortlisted articles will be then be obtained and evaluated against inclusion and exclusion criteria. Any disagreement between the two authors (MWBZ and JY) will be resolved through a discussion with the third author (GS). An electronic form will be used to record reasons for inclusion and exclusion of articles. This systematic review protocol will adhere to reporting guidelines of Preferred Reporting Items for Systematic Reviews and Meta-Analysis Protocols (PRISMA-P) [28]. Figure 1 shows a sample of the PRISMA diagram that we will include in the final protocol.

The following data and information will be extracted from each article, cross-checked by the second author, and recorded on a standardized electronic data collation form:

1. Publication details: author(s) and study year
2. Study design and methodology: sample size (intervention and control group), characteristics of the sample (mean age and percentage of males and females), method by which participants were recruited; main psychiatric diagnosis for participants; how psychiatric diagnosis was made (clinical versus questionnaire)
3. Cognitive bias task utilized
4. Outcomes of interest: effectiveness of bias modification (effect sizes, either Cohen *d* or Hedge G, if reported) and secondary outcomes reported (for example, scores on questionnaires for anxiety or depressive symptoms, craving scores, mean time to relapse, and total duration of abstinence).

### Quality Assessment

For risk of bias assessment, the Cochrane Risk of Bias tool [29] will be used for grading. Studies will be evaluated for risk of selection bias, performance bias, detection bias, attrition bias, and reporting bias. Risk of bias assessment will be conducted by the author (MZ) and then verified by the author (JY). Any disagreements will be resolved through a discussion with the third author (GS).

### Strategy for Data Integration and Synthesis

A narrative synthesis of all relevant articles will be conducted. Results will be organized by different types of psychiatric disorders, for example, addictive, depressive, and anxiety. The number of studies reporting effectiveness in each psychiatric condition will be stated. Results about secondary outcomes will be summarized.

Meta-analysis will be considered only if there are sufficient articles in a domain for quantitative statistical analysis. For the meta-analytic study, statistical analysis will be performed using Comprehensive Meta-Analysis Version 2.0 (BioStats, NJ, USA) based on the random effects model. The random effects model will be used because it assumes varying effect size between studies and because of underlying differences in study design and heterogeneity of the sampled population. Statistical analysis aims to compute pooled effect size to determine the clinical efficacy of cognitive bias modification for each different disorder. The analysis also aims to identify potential moderators that could account for heterogeneity in the effect size computed. Between-study heterogeneity will be assessed with the I^2^ statistic, which describes the percentage of variability among effect estimates beyond that expected by chance. As a reference, I^2^ values of 25% are considered low, 50% moderate, and 75% high in heterogeneity. Meta-regression analysis will be performed to determine if continuous variables such as mean age and proportion of males or females affect heterogeneity in the pooled effect size of cognitive bias modification for a psychiatric disorder. Subgroup analysis will be undertaken to investigate effects of variables (method of psychiatric diagnosis, cognitive bias task utilized) on the effect size obtained. For meta-analysis, Egger regression test will be conducted to determine if publication bias is present. If there is significant publication bias, the classic fail-safe test will be performed to determine the number of missing studies that will be required for the *P* value of publication bias to be higher than .05.

### Ethics and Dissemination

Ethical approval for this protocol and planned systematic review was not required. Results synthesized would be disseminated by means of conference presentation or published works in peer-reviewed journals.

### Protocol Registration

The protocol for this planned systematic review has been registered with PROSPERO in 2017 (CRD42017074754).

## Results

We have screened 2674 unique articles identified from all the selected databases using the predefined search strategy. We expect that the review will be completed 12 months from the publication of this protocol. 

## Discussion

Based on our knowledge, there have been systematic reviews as well as meta-analyses published reporting the efficacy of cognitive bias modification for substance use disorders and for other psychiatric disorders. This review is timely because it will expand on findings of prior reviews. In addition, the synthesis of existing evidence will help inform researchers and clinicians as to whether Web-based cognitive bias interventions are effective and if so, for which psychiatric disorder(s). Examining the effectiveness of Web-based interventions is undoubtedly crucial, given that they reduce geographical barriers associated with receiving therapy and reduce the need for a therapist [18] [19]. Web-based cognitive bias modification, if proven effective, could help reduce overall cost associated with receiving psychological interventions.

## Abbreviations

PRISMA: Preferred Reporting Items for Systematic Review and Meta-analyses
